# Single-shot femtosecond bulk micromachining of silicon with mid-IR tightly focused beams

**DOI:** 10.1038/s41598-022-11501-4

**Published:** 2022-05-07

**Authors:** Evgenii Mareev, Andrey Pushkin, Ekaterina Migal, Kirill Lvov, Sergey Stremoukhov, Fedor Potemkin

**Affiliations:** Faculty of Physics, M. V. Lomonosov Moscow State University, Leninskie Gory bld. 1/2, 119991 Moscow, Russia

**Keywords:** Laser material processing, Mid-infrared photonics, Phase transitions and critical phenomena

## Abstract

Being the second most abundant element on earth after oxygen, silicon remains the working horse for key technologies for the years. Novel photonics platform for high-speed data transfer and optical memory demands higher flexibility of the silicon modification, including on-chip and in-bulk inscription regimes. These are deepness, three-dimensionality, controllability of sizes and morphology of created modifications. Mid-IR (beyond 4 µm) ultrafast lasers provide the required control for all these parameters not only on the surface (as in the case of the lithographic techniques), but also inside the bulk of the semiconductor, paving the way to an unprecedented variety of properties that can be encoded via such an excitation. We estimated the deposited energy density as 6 kJ cm^−3^ inside silicon under tight focusing of mid-IR femtosecond laser radiation, which exceeds the threshold value determined by the specific heat of fusion (~ 4 kJ cm^−3^). In such a regime, we successfully performed single-pulse silicon microstructuring. Using third-harmonic and near-IR microscopy, and molecular dynamics, we demonstrated that there is a low-density region in the center of a micromodification, surrounded by a “ring” with higher density, that could be an evidence of its micro-void structure. The formation of created micromodification could be controlled in situ using third-harmonic generation microscopy. The numerical simulation indicates that single-shot damage becomes possible due to electrons heating in the conduction band up to 8 eV (mean thermal energy) and the subsequent generation of microplasma with an overcritical density of 8.5 × 10^21^ cm^−3^. These results promise to be the foundation of a new approach of deep three-dimensional single-shot bulk micromachining of silicon.

## Introduction

The silicon (Si) is one of the most important materials for modern electronics and photonics. However, due to low bandgap (~ 1.1 eV) and high refractive index (~ 3.3), it is challenging to perform the three-dimensional (3D) femtosecond micromachining of its volume^1,2^. The two-photon absorption^2^, aberrations induced by refractive index mismatch^3^, and plasma delocalization in the pre-focal volume^4^ drastically spread the energy of the femtosecond pulse across a large volume and drop the deposited energy density below the threshold of micromodification formation^5^. Nowadays, there is a race for creating an efficient way for femtosecond micromachining of Si. In contradistinction to the nanosecond laser pulses, which were successfully used for Si micromachining^6^, the femtosecond pulses engage for lower thermal impact on the material and, as a result, a better resolution and controllability^7^. The first micromachining of Si by femtosecond laser pulses was achieved using solid immersion^8^; extreme (NA > 3) focusing conditions are fulfilled under such a geometry. However, this approach is complicated for further technological applications. The alternative way is to use seed electrons created by the prior femtosecond pulse^9^ or by pico- or nanosecond pedestal^10^. In this case, the localization of the laser pulse energy is achieved by the first low-energy laser pulse, which, thanks to low intensities, avoids plasma delocalization^11^. Another approach is based on getting away from two-photon absorption using sub-ps and ps laser pulses at a wavelength from 1.9 to 2.6 μm^12–15^. In addition, a longer pulse duration decreases the intensity and plasma electron density, which allows avoiding strong absorption in the pre-focal volume. Nevertheless, the degree of multiphoton order (the ratio of the photon energy to the bandgap) stays comparably low (~ 2–3), leading to the plasma screening of the laser pulse even at sub-μJ levels.

Shifting central wavelength of a driving pulse into the mid-IR can make the process of photoionization^16^ dominant in semiconductors, avoid two- and three-photon absorption, and create single-shot femtosecond micromodification in a bulk Si for a broad range of energies. In this paper, we demonstrate that the tightly focused mid-IR femtosecond pulses are capable of micromodification creation due to overcoming deposited energy density threshold, determined by the latent heat of fusion (about 4 kJ cm^−3^). The numerical simulations show that it becomes possible due to electrons heating in the conduction band up to values much higher than the bandgap value and the subsequent generation of microplasma with an overcritical density.

## Results and discussion

### Deposited energy density (DED) retrieving

**Figure 1:**
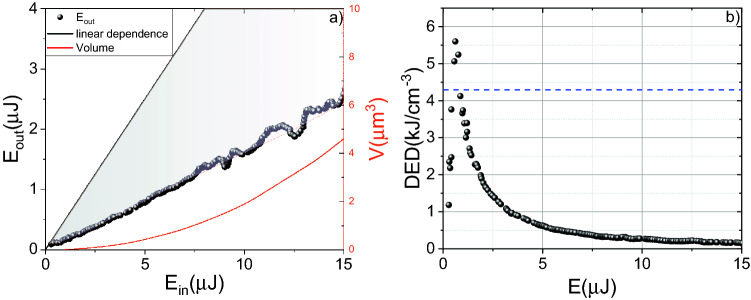
(**a**) Energy dependence of nonlinear transmission (black dots) and interaction volume (solid red line). The shaded area shows the absorbed energy region. (**b**) Dependence of DED on the laser pulse energy. The dotted line shows the threshold of micromodification formation.

The silicon is an indirect bandgap semiconductor with a bandgap of about 1.1 eV (at 300 K)^17^. In Si irradiated with femtosecond laser pulses the conduction band initially has to be filled with free electrons from the valence band. These electrons can absorb photons via inverse Breamsstrauhlung absorption and ignite the impact ionization process^18^. Then the electrons via electron–phonon interaction transfer the energy to the lattice. In particular, they heat the lattice^19^ and induce GPa pressures^20^. The amount of transferred energy strongly depends not only on the plasma electron density but also on the electron temperature^16^. It also can be described in the framework of the deposited energy density (DED)^21^. The threshold (necessary for micromodification formation) DED value can be estimated as latent heat of fusion in Si ~ 4.2 kJ cm^−3^^22^. The deposited energy density is defined as DED = E_abs_/V_int_, where E_abs_ is the absorbed energy, and V_int_ is the volume in which this energy is absorbed (interaction volume). To determine DED both parameters should be measured in the experiment. While absorbed energy is easy to measure (using nonlinear absorption measurements—see “Methods”), the volume measurement in the case of mid-IR excitation is challenging. Since the 3D propagation imaging (fluence) technique^8,23^ that we have previously proposed for the near-IR radiation is poorly applicable due to the low sensitivity of the camera (Pyrocam Spiricon III) available for this work (about tens of μJ), V_int_ was estimated only for higher energies. A femtosecond pulse (4.6 μm, energy up to 3 mJ, time duration ~ 160 fs, frequency 10 Hz) was focused into the air to obtain V_int_ values for low energies, creating a plasma. The size of this plasma’s luminescence at different energies was recorded using a CCD camera (MindVision) with a spatial resolution of the order of 1 pixel per μm. A power-law function was used for approximation the dependence of plasma size on energy, that is non-rigorous extrapolation of the interaction volume for simplification. Under tight focusing of mid-IR laser radiation into the silicon, V_int_ increases; thereby, we compared the obtained V_int_ values in silicon and in the air at a laser energy of 50 μJ. In silicon, V_int_ is about 18 times higher than in air (the difference in diameter is 2.2 times and 3.7-fold in length). Assuming that the volume dependence on energy in silicon does not differ from its behavior in air, we estimated V_int_ (see Fig. 1a) and calculated the DED (see Fig. 1b). Moreover, we verified the method by measuring the plasma luminescence (~ 900 nm) profile in Si as proposed in^24^. The plasma luminescence was registered by CMOS camera (DMK 38GX304, Imaging Source), each profile is a sum of 60 images obtained with minimal exposure ~ 2 μs in the trigger mode. Si has absorbance of ~ 100 cm^−1^ at this wavelength, and to obtain this signal we focused laser pulse near the sample’s surface. The diameter and the length of plasma is in a good coincidence (~ 10% error) with data obtained by the method described above. The CCD camera was used to obtain the plasma profile in air.

**Figure 2:**
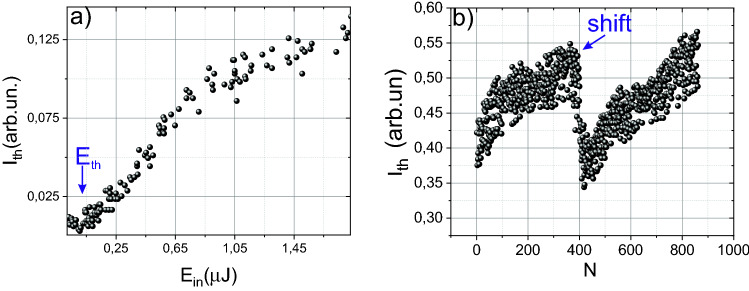
(**a**) Dependence of the third harmonic generation efficiency on the laser pulse energy. The arrow indicates the plasma formation threshold. The sample is moved from pulse to pulse. (**b**) Dependence of the third harmonic signal on the number of laser pulses. The arrow indicates the moment of the silicon sample shift.

Nevertheless, we obtained the dependence of the absorbed energy and the interaction volume on the laser pulse energy and retrieved the DED (see Fig. 1a). There is a clearly expressed maximum in the DED dependence on laser pulse energy, corresponding to 1.3 μJ. In technology, it is extremely important to control the process in situ. Thereby in our experiments, the threshold of micromodification formation was determined online from third-harmonic measurement. Under tight focusing due to the destructive interference before and behind the focal plane, the efficiency of non-synchronous third-harmonic generation would tend to zero in the undamaged material^25^. But the plasma generation or micromodification formation breaks the conditions of such interference^26^. When the sample is not shifted from pulse to pulse, the formation of the micromodification from single pulse will lead to the generation of the third harmonic by subsequent pulses (see “Methods”). The abrupt increase of the third harmonic signal serves as an in situ indicator of the micromodification formation (see Fig. 2a). This threshold is about 1.1 μJ (see “Methods”). Below this threshold, there is no increase of the third harmonic signal. To determine this threshold, we consistently increased the laser pulse energy until the specific picture, represented in Fig. 2b, is occurred. The plasma formation threshold was obtained in the scheme similar to transmittance measurement, except we registered the third harmonic signal instead of the transmitted pulse energy (the sample was shifted from pulse to pulse). The threshold for plasma formation is about 200 nJ (see Fig. 2a), determined as energy corresponding to an increase of the third harmonic generation efficiency.

Thereby we prove that overcoming the plasma formation threshold is not a sufficient condition for micromodification formation. Due to a quite narrow bandgap (hω ~ 1 eV), only at n_e_ ~ 1/2n_a_ ~ 2.5 × 10^22^ cm^−3^, the DED would be close to a micromodification formation threshold (it can be estimated from specific heat of fusion ~ 4.2 kJ cm^−3^). It should also be noted that for high energies (above 3 μJ), due to a substantial increase in the V_int_, obtained experimental values of the DED rise to be microscopic and becomes macroscopic, i.e., it is volume-averaged. Therefore, a drop in DED below the threshold will not indicate the absence of micromodification, and the local values of DED remain higher than the threshold. However, the presence of a pronounced maximum means that the maximal efficiency of micromodification formation is achieved. It is also worth noting that these experiments were carried out for the focal position at a depth of 125 μm from the sample surface. The change in this parameter will significantly affect the amount of delivered energy due to aberrations arising from the tight focusing of laser radiation on the flat input surface of the silicon sample^3^.

### Retrieving of the micromodification size

**Figure 3:**
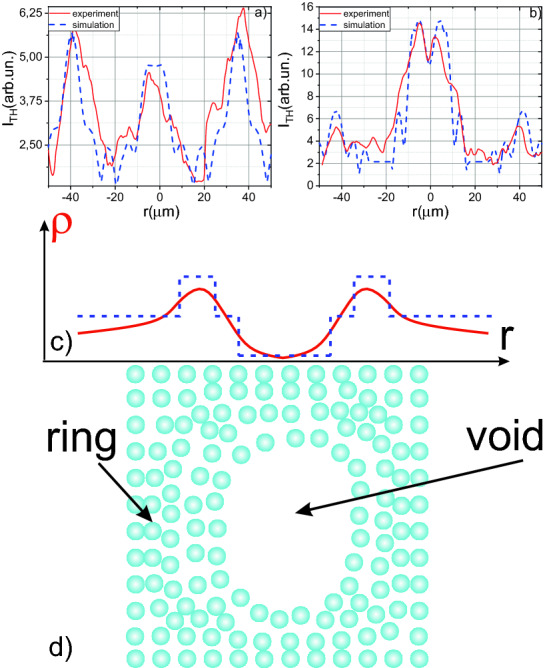
Dependence of the third harmonic intensity on the radial shift from the center for micromodification with (**a**) 3.4 μm and (**b**) 7.2 μm in diameter. (**c**) Radial profile of density (solid red curve) in microvoid and used for the profile of third-order nonlinear susceptibility χ^(3)^ in the XY-plane (z is an optical axis) used in the simulations. (**d**) Visualization of microvoid profile in XY-plane.

The next series of experiments were aimed to determine the dimensions of the created micromodifications and their dependence on the laser pulse energy. In addition to post-mortem microscopy, we also use in-situ diagnostics of micromodification formation based on the third harmonic (~ 1.5 μm) generation. Under tight focusing, the third harmonic is generated on the medium inhomogeneities^27^. Thus, the creation of micromodification will lead to the appearance of the third harmonic signal, which serves as an indicator of successful bulk micromachining (see Fig. 2b). We scanned the created micromodifications using the third harmonic imaging technique^27^. When the sample with micromodification to be inscribed in bulk is shifted along the direction perpendicular to the optical axis, a peak in the third harmonic signal corresponds to the center of the micromodification. In addition, there is a second peak at a distance of about 40–50 μm from the center. Such a third harmonic profile arises from the morphology of micromodification: void is located in the center and surrounded by the ring of compressed material with a higher density, created by the shock wave (see Fig. 3d). Thereby in analogy with dielectrics, that micromodification has a micro-void morphology^28^. We also simulated the process of micromodification formation in Si using molecular dynamics and two-temperature model (see section Molecular dynamics). The diameter of the ring at energies above 2 μJ is essentially independent on the laser pulse energy. Assuming that the fraction of energy transmitted to the shock wave linearly depends on the DED we could postulate that DED saturates at high energies that correspond to the drop of volume-averaged DED from Fig. 1.

We also simulated the third harmonic profile using the assumption that third-order nonlinear susceptibility has a profile shown in Fig. 3c (dotted line). The simulation coincides with experimental data and successfully reconstructs the main features in the third harmonic profile. By carefully analyzing the dependence of the third harmonic signal on the coordinate in the XY plane (z is an optical axis), it can be noted that the third harmonic is generated at both boundaries of the central part of the micromodification. At a smaller size of micromodification, these peaks merge into one peak^28^ (see Fig. 3a,b).

**Figure 4:**
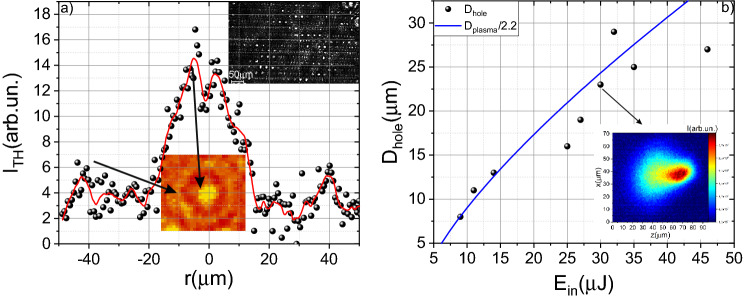
(**a**) Micromodification profile obtained by third harmonic imaging. The energy of the laser pulse is 1.1 μJ. The insets show single micromodification as well as an array of created micromodifications. (**b**) Dependence of the micromodification diameter on the laser pulse energy. The blue line shows the dependence of the plasma diameter in the air (divided by a factor of 2.2) on the laser pulse energy. The inset demonstrates the plasma luminescence profile.

The ring around the central part, formed by the shock wave, leads to a smaller jump in third-order nonlinear susceptibility χ^(3)^ at the center of micromodification. The central part of the micromodification is essentially a void; thereby, a significant jump in nonlinearity is observed at the micromodification boundary (see “Methods”). While passing along the ring, the density changes significantly less, which leads to a lower amplitude of the third harmonic signal. The sizes of micromodifications obtained by the third harmonic generation technique fully correspond to the results of optical microscopy (see Fig. 4a). Also, the validity of the method used to determine the DED is confirmed by the fact that when we superimpose the dependence of the plasma diameter on energy divided by a factor of 2.2 on the graph of the micromodification diameter versus energy, they coincide with reasonable accuracy (see Fig. 4b). As shown in Fig. 4, the micromodification diameter increases with increasing energy due to an increase in the interaction volume. In our experiments, the minimum observed micromodification diameter was 3 µm, and the maximum registered size was 28 µm. The diameter of the created micromodifications slightly fluctuates from pulse to pulse as could be seen from the inset on Fig. 4a due to strong dependence on laser pulse energy (RMS ~ 7%). The process of the micromodification formation after laser impact is revealed in the Molecular dynamics Section further in the text.

It should be noted that since the radiation at a wavelength of 4.6 μm is generated using CPA (chirped pulse amplification), the laser pulse may contain a pico- and nanosecond pedestal, which, following^10^, may increase the probability of the damage. We measured the pulse contrast of the laser system using third-order cross-correlator. Its dynamical range is about six orders of magnitude. We detected no post- or pre-pulses. In addition, we can estimate the nanosecond intensity contrast, which is better than 10^6^. It corresponds to ~ 10^2^-energy contrast. For the optimal laser pulse energies ~ 1 μJ, the energy of the nanosecond pedestal would be less than 10 nJ, that is less than nanosecond threshold of Si damage^6^.

### Numerical calculation of energy deposition

To confirm the single-shot femtosecond bulk micromachining of Si and reveal possible physical mechanisms leading to this goal, we support experimental investigations with numerical simulation of free electron density dynamics and deposited energy density. Our findings show quantitative coincidence of theoretical results with experiments and estimate both achieved free electron density and thermal energy.

The micromodification formation inside the bulk of the transparent material is determined by the laser energy density, which is deposited to the material through excitation of the electronic subsystem. Nowadays, electron dynamics in the valence and conduction bands are frequently calculated with the Multiple Rate Equations (MRE)^29^, which are naturally emerging from calculations based on the kinetic Boltzmann equation^30^ and account for the discrete distribution of electron energy in the conduction band, thus delaying the impact ionization concerning the field ionization. But it is worth noting that in MRE, the probability of impact ionization α is much larger than the one-photon absorption probability W_1pt_. It means that there is only one energy level with the energy above a critical value (which is needed to launch the impact ionization process). Once an electron reaches the critical level, it immediately creates one more electron in the conduction band and decreases to the lower energy level. Therefore, electrons are limited in their kinetic energy by the critical energy: ε_cr_ = 1.5 × (E_g_ + U_p_) = 1.5 × (E_g_ + 7.5 ∙ I[TW cm^−2^] eV) ~ 2.8 eV for the laser intensity 10^11^ W cm^−2^, which is typical one reached inside Si (here U_p_ is the ponderomotive energy). But in mid-IR, the one-photon absorption probability becomes comparable with the probability of impact ionization. The former reads as^22^:

1 $${W}_{1pt}=\frac{{U}_{p}}{\hslash \omega }\cdot \frac{{\nu }_{c}}{1+{\left({\nu }_{c}/\omega \right)}^{2}}\approx \frac{{U}_{p}}{\hslash \omega }\cdot \frac{\pi {\varepsilon }_{0}}{3{e}^{2}}\sqrt{\frac{2}{{m}_{eff}}}{{\varepsilon }_{k}}^{1.5}=2.5\cdot I\left[\frac{TW}{c{m}^{2}}\right]\cdot {{\varepsilon }_{k}\left[eV\right]}^{1.5} f{s}^{-1}$$

For the laser intensity I = 10^11^ W cm^−2^ and mean kinetic energy of electrons ε_k_ = 0.5·ε_cr_ = 1.4 eV the one-photon absorption probability reaches the value of 0.4 fs^−1^, which is comparable with the typical value 1 fs^−1^ of the impact ionization probability^31^. This result indicates that energy levels above the critical level should be considered in the MRE. To reduce the computational complexity of such electron dynamics calculations, we introduce an averaged energy level above the critical level (see details in “Methods”). This part of electrons is heated by laser radiation and participates in full in the impact ionization mechanism.

**Figure 5:**
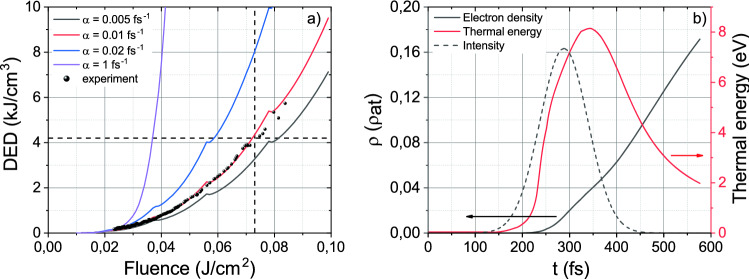
(**a**) Dependence of deposited energy density (DED) for the different probability of impact ionization α on fluence. Black points indicate the experimental values of DED. The horizontal dashed line marks the DED threshold, which is needed for Si crystal lattice melting and the vertical dashed line marks the fluence threshold, which is needed to achieve the DED threshold value. (**b**) Free electron density and mean thermal energy during the laser pulse action. The pulse intensity profile is shown as the dashed line. Pulse fluence corresponds to the threshold value of 0.073 J/cm^2^.

Since the probability of impact ionization α is generally set heuristically^29,31^, we analyze electron dynamics with the increase of laser fluence for the wide range of α from 0.005 to 1 fs^−1^ (some of them are presented in Fig. 5). For the high values of the impact ionization probability, electron avalanche rapidly progresses once electrons are heated up to the critical energy. Deposited energy density, therefore, inevitably grows up since it is proportional to the free electron density. For the low values of the impact ionization probability, much more electrons populate the averaged energy level introduced to the standard MRE model and, as a result, demonstrate more smooth growth with laser fluence increase. Retrieving the laser fluences achieved in the experiment, we show experimental results as black dots in Fig. 5. The deposited energy density reveals power dependence on the laser fluence with a degree of 2.8, corresponding to the probability of impact ionization α = 0.01 fs^−1^. It agrees with the value proposed in some works^16^.

The dashed horizontal line in Fig. 5a indicates the DED threshold of Si crystal lattice melting which can be simply estimated as L·ρ_m_/μ = 4.2 kJ cm^−3^ (where L is the latent heat of fusion, ρ_m_ is the mass density, μ is the molar mass). According to numerical calculations, this threshold value is achieved for laser fluences higher than 0.073 J cm^−2^ (dashed vertical line in Fig. 5a).

It is worth noting that DED is not only defined by achieved electron density (due to photoionization), but it also severely relates to thermal energy that electrons accumulate in the conduction band under the laser field impact. As is mentioned above, in mid-IR the one-photon absorption probability reaches significantly higher values as compared to the visible wavelength range. Therefore, electrons in the conduction band undergo considerable heating before impact ionization (see Fig. 5b). Electron means thermal energy is slightly higher than 8 eV for the laser fluence of 0.073 J cm^−2^ which is more than 7 times higher than the Si bandgap (1.12 eV). It indicates that the contribution of electrons heating into DED is more than that of field ionization. The rise in electron thermal energy corresponds to the central part of a pulse where the intensity and therefore the one-photon absorption rate is the highest. At the pulse tail due to lower intensity, the one-photon absorption rate decreases down to the impact rate, and the process of impact ionization effectively occurs giving rise to the electron density. To the pulse end free electron density achieves the value of 0.17·ρ_at_ = 8.5 × 10^21^ cm^3^, which is approximately 170 times larger than the critical density (ρ_cr_ = 5 × 10^19^ cm^−3^ for λ = 4.6 µm).

### Molecular dynamics simulation

The Two-Temperature Model (TTM) describes a nonequilibrium state between laser-induced electrons and the lattice, formed when the sample is irradiated by an ultrashort laser pulse^32^. The time required to establish equilibrium in the electron gas is much less than the time required to establish equilibrium between the electron and the lattice phonons. Thus, the sample (Si in our case) can be considered as composed of interacting subsystems consisting of electrons and phonons. The hot thermalized electron gas relaxes to the bath phonons with relatively slow electron–phonon interactions^33^. On the other side the mechanical post-effects, such as shock waves and phase transitions, could be successfully reproduced by molecular dynamics^34^. Combining these approaches gives the opportunity to retrieve lattice dynamics after interaction of femtosecond laser pulse with Si sample. We used an approach proposed in^35^. The details of numerical simulation are presented in the “Methods” section.

**Figure 6:**
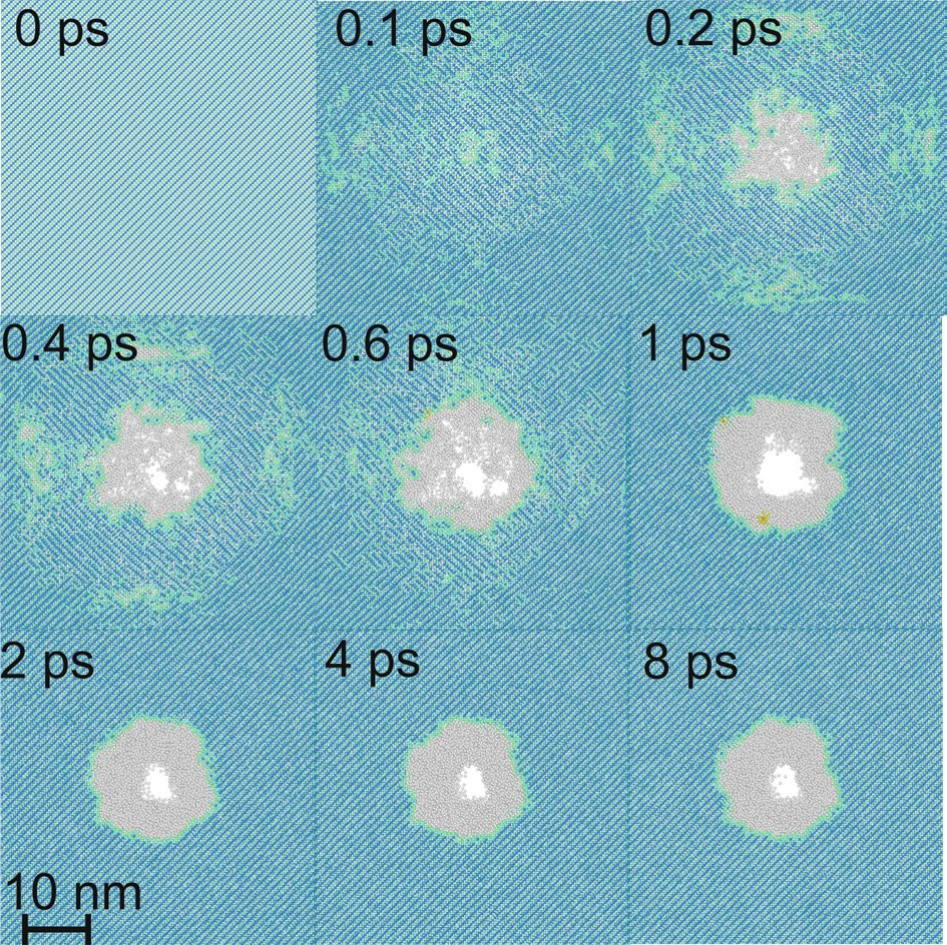
Evolution of laser-induced microvoid formation (10 A slice along 001 is shown). Blue color indicates Si in a-diamond phase, white in an amorphous, green is an intermediate case.

The performed simulation revealed the main phases of microvoid formation in Si (see Fig. 6). Initially, rapid heating of lattice through electron–phonon interactions in combination with a blast force arise from the interaction of electrons with ions leads to the amorphization of Si in the center of laser irradiation, and a non-zero radial velocity component is generated. It leads to the formation of microvoid “seeds” at 0.2–0.4 ps. Then in about 1 ps the microvoid is formed, and at this moment, the shock wave (ring across the microvoid) leaves the simulation area. Then the compressed material in the “ring” starts to expand to the void. However, when the temperature of the atoms becomes lower than the melting point (~ 2 ps) the final structure is formed. The resulting structure qualitatively coincides with experimental data. Depending on laser pulse parameters, no microvoid could be formed. In this case, only amorphous Si in the center is observed, however the “ring” formation is always followed by microvoid generation. The full evolution is presented in “Supplementary material”.

## Conclusion

We, for the first time, demonstrate the possibility of creating micromodification under tight focusing (NA = 0.86 f = 1.84 mm) of mid-IR femtosecond radiation (energy ~ few μJ, pulse duration ~ 160 fs, the wavelength is 4.6 μm) into the bulk Si [001]. Micromodifications have a size from 3 to 30 μm and a micro-void morphology: a void is surrounded by a ring of carried mass. It indicates a high localization of the absorbed energy in the microvolume, which is confirmed in the experiment by the measured DED values (> 6 kJ cm^−3^). The process of plasma generation and the formation of micromodifications were monitored by the third harmonic imaging, including in-situ diagnostics.

Numerical simulation of the conduction band population dynamics is performed with extended Multiple Rate Equation model allowing electrons to be heated above the critical energy of impact ionization. By finding the coincidence between theoretical consideration and experimental results, the value 0.01 fs^−1^ of the impact ionization probability is determined. Numerical studies allow us to assert that high deposited energy density is related with electrons heating in the conduction band up to 8 eV and that for the implementation of micromachining, it is necessary to create plasma densities of 8.5 × 10^21^ cm^−3^, which is higher than the critical density.

## Methods

### Nonlinear transmission measurements

A femtosecond laser system based on Fe^2+^:ZnSe CPA (10 Hz, 160 fs, 3 mJ) was used in this work. A lens with a numerical aperture of NA = 0.86 (Thorlabs 390037-E) tightly focuses a laser beam into the bulk of silicon. A laser spark was ignited at the silicon boundary before the experiment to avoid surface damage due to laser ablation. Further, the sample was shifted along the z-axis (optical axis) so that the focus was at a depth of about 125 µm. The sample was placed on a motorized three-axis translation stage; the movement was carried out at 0.625 µm per second speed. In this regime, a single-pulse interaction was achieved. A calibrated PbSe detector monitored the laser pulse energy, the tiny part of laser radiation was reflected on the detector by a CaF_2_ plate. A silicon polarizer (Thorlabs WP25M-IRA) was used to vary the energy. The maximum energy used in the experiment reached 80 μJ. The radiation transmitted through the sample and its third harmonic was collimated using a lens with a numerical aperture of 0.45 (Thorlabs 390028-D). The pulse’s energy passed through the sample (2 × 2 × 2 mm) was monitored by a PbSe detector, and a Ge photodetector registered the energy of the third harmonic signal. Varying the laser pulse energy while simultaneously recording the radiation transmitted through the sample and its third harmonic, we plotted the nonlinear transmission curves and the dependence of the third harmonic signal on the laser pulse energy^28^.

### Third harmonic imaging

**Figure 7:**
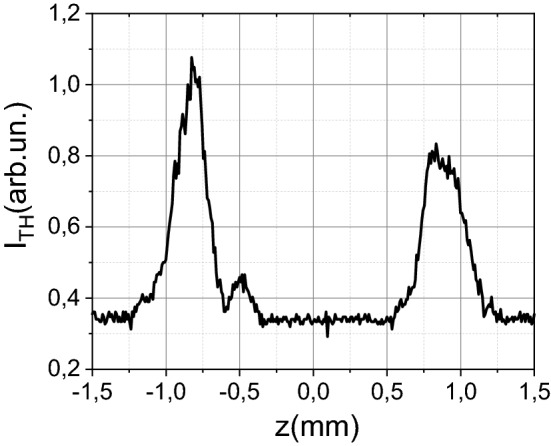
(**a**) The third harmonic profile during the movement of the sample along the optical axis.

In the framework of nonlinear transmission measurements, it is impossible to determine the threshold of micromodification formation under single-pulse impact. Moreover, it is difficult to determine the plasma formation threshold. Under tight focusing of mid-IR femtosecond laser radiation into the isotropic medium, the third harmonic (~ 1.5 μm, Si is transparent for this wavelength) signal will be equal to zero due to destructive interference of laser radiation before and after the focal plane^27^. Though Si is not isotropic, the signal of the third harmonic generated due to its inherent anisotropy is about one order lower than the third harmonic induced on the laser-induced plasma or micromodifications. The appearance of any inhomogeneities violates the conditions of destructive interference, which leads to the generation of the third harmonic. In our case, micromodification acts as an inhomogeneity. As already shown in our previous works^27,36^, this technique makes it possible to reconstruct both the dimensions of the micromodification and the dimensions of the laser-induced plasma in dielectrics. Moreover, moving the sample along the optical axes leads to the third harmonic generation on its boundaries, see Fig. 7.

In the experiments, if we did not shift the sample, the subsequent pulses would generate the third harmonic on the micromodification created by previous one. The shift of the sample would lead to the drastic drop of the third harmonic signal. The third harmonic signal increased during the growth of damage size. We use this dependence for the in-situ control of micromodification formation. The example of the third harmonic dependence on the number of pulses is presented in Fig. 2b. With this method it is possible to determine the threshold of micromodification formation in a multi-pulse interaction regime. Moreover, we used the third harmonic imaging for mapping the micromodification. In this case, a pump pulse created micromodification; further, the laser radiation was blocked with the beam dumper, and the energy decreased (below the threshold of micromodification formation). After that, the sample was moved perpendicular to the axis of the laser radiation propagation. Thus, the profile of the third harmonic signal was recorded. In this dependence, both the boundaries of micromodification and the ring of the removal mass, which appears during the propagation of a shock wave, stand out^28^ (see Figs. 3, 4). This profile is typical for microvoids, as we have shown in the case of dielectrics^28^. We also performed simulations of the third harmonic generation on the micromodifications. We used the same approach as in^36^. The efficiency of the third harmonic generation for the given coordinates (z_0_, y_0_) can be expressed as^27^:

2 $$\eta (z_{0} ,y_{0} )\sim \frac{{[\chi^{(3)} ]^{2} d_{0}^{3} E^{2} }}{{L_{0} \cdot n_{3\omega }^{{}} }} \cdot \left| {\int\limits_{{z_{0} - L_{0} }}^{{z_{0} + L_{0} }} {\int\limits_{ - 1}^{1} {\exp ( - \xi^{2} /2)} \frac{{\exp (i\Delta k(z - z_{0} ))}}{{(1 - {{2i(z - z_{0} )} \mathord{\left/ {\vphantom {{2i(z - z_{0} )} {L_{0} }}} \right. \kern-\nulldelimiterspace} {L_{0} }})^{2} }}} dzd\xi } \right|,$$

where ξ = (y − y_0_)/w_0_, E denotes laser pulse energy, d_0_ is the laser waist diameter (1/e), L_0_ is the beam waist length (1/e) ~ 16 μm, Δk = k_3ω _− 3k_ω_ (corresponds to the coherent length ~ 4 μm) the phase mismatch between the third harmonic k_3ω_ and fundamental frequency k_ω_, χ^(3)^ is the third-order susceptibility. For simulation, we used the χ^(3)^ profile presented in Fig. 3c (dotted line). We varied the diameter of the microvoid and the “ring” until the coincidence with the experiment, as is shown in Fig. 3a. For small (less than 4 µm in size) microvoids, there is only one peak in the center. The increase of the microvoid’s size leads to the appearance of two peaks in the center.

### IR microscopy

To verify this technique in semiconductors, we performed microscopy of a micromodifications array created in the bulk of the sample. The radiation of a femtosecond Cr: Forsterite laser (10 Hz, 3 mJ, 100 fs) was applied as illumination. Using a microscope objective with a numerical aperture of 0.4 (LOMO), the image was transferred to the matrix of a silicon CCD camera (MindVision). It should be noted that this matrix is sensitive to near-infrared (1240 nm) radiation due to two-photon absorption^4^. The spatial resolution of the microscopy was about 2 µm pixel^−1^.

### Simulation of free electron density dynamics

To describe free electron density ρ evolution, we propose an extension to the original Multiple Rate Equation (MRE)^29^. In the MRE, an assumption that the impact probability is much more significant than the one-photon absorption α » W_1pt_ is made, thus limiting the maximal free electron energy by the critical value ε_cr_ = 1.5 × (E_g_ + U_p_) = 1.5 × (E_g_ + 7.5 × I[T.W. cm^−2^] eV), where U_p_ is the ponderomotive energy. But in mid-IR, the one-photon absorption probability becomes comparable with the probability of impact ionization. The former reads as^22^:

3 $${W}_{1pt}=\frac{{U}_{p}}{\hslash \omega }\cdot \frac{{\nu }_{c}}{1+{\left({\nu }_{c}/\omega \right)}^{2}}\approx \frac{{U}_{p}}{\hslash \omega }\cdot \frac{\pi {\varepsilon }_{0}}{3{e}^{2}}\sqrt{\frac{2}{{m}_{eff}}}{{\varepsilon }_{k}}^{1.5}=2.5\cdot I\left[\frac{TW}{c{m}^{2}}\right]\cdot {{\varepsilon }_{k}\left[eV\right]}^{1.5} f{s}^{-1}.$$

For the laser intensity I = 10^11^ W cm^−2^ and mean kinetic energy of electrons ε_k_ = 0.5 × ε_cr_ = 1.4 eV the one-photon absorption probability reaches the value of 0.4 fs^−1^, which is comparable with the typical value 1 fs^−1^ of the impact ionization probability^31^. This result indicates that energy levels above the critical level should be taken into account in the MRE. Therefore we introduce additional averaged energy level above the critical level and its population n_a_ and energy density u_a_ changes as:

4 $$\frac{\partial {n}_{a}}{\partial t}={W}_{1pt}\left(t\right){n}_{cr}-\alpha {n}_{a},$$

5 $$\frac{\partial {u}_{a}}{\partial t}={W}_{1pt}\left(t\right){n}_{cr}\left({\varepsilon }_{cr}+\hslash {\omega }_{0}\right)+{W}_{1pt}\left(t\right){n}_{a}\hslash {\omega }_{0}-\alpha {n}_{a}{u}_{a},$$

where n_cr_ is the population of the critical level, ω_0_ is the laser frequency. The first term on the right-hand side describes energy accumulation by transitions from the critical level to the averaged level, the second one—energy acquisition due to one-photon absorption, and the last one—energy decrease caused by the impact ionization process.

The rest energy u_a_/n_a_ − ε_cr_ after the impact ionization of an electron in the introduced averaged level has to be divided between two electrons at the level k = 0.5 × (u_a_/n_a_ − ε_cr_)/ħω_0_. Therefore the population evolution of this energy level also has to be modified:

6 $$\frac{\partial {n}_{k}}{\partial t}={W}_{1pt}(t)({n}_{k-1}-{n}_{k})+2\alpha {n}_{a}$$

The probability of one-photon absorption is not a constant but is determined by the collision frequency ν_c_ and the laser intensity I:

7 $${W}_{1pt}\left(t\right)=\frac{{U}_{p}}{\hslash {\omega }_{0}}\cdot \frac{{\nu }_{c}\left(t\right)}{1+{\left({\nu }_{c}\left(t\right)/{\omega }_{0}\right)}^{2}}, { U}_{p}=\frac{{e}^{2}I}{c{n}_{0}{\varepsilon }_{0}{m}_{e}{\omega }_{0}^{2}},$$

Collision rate ν_c_ is not a constant and is determined by the mean kinetic energy of free electrons ε_K_^22^:

8 $${\nu }_{c}=\frac{\pi {\varepsilon }_{0}}{3{e}^{2}}\sqrt{\frac{2}{{m}_{e}}}{\varepsilon }_{K}^{1.5},$$

where e is the electron charge, m_e_ is the electron effective mass in the conduction band. The mean kinetic energy is found as a sum of all energy levels in the conduction band:

9 $${\varepsilon }_{K}=\frac{1}{\rho }\sum_{m=0}^{{n}_{cr}}{n}_{m}m\hslash {\omega }_{0}+{\varepsilon }_{a}, \rho =\sum_{m=0}^{{n}_{cr}}{n}_{m}+{n}_{a}$$

The model presented above gives a path to calculate evolution with time of the free electron density in the conduction band and collision frequency and one-photon absorption probability. After the laser pulse impact, one can retrieve deposited energy density as an integral over time of energy density rate transmitted from the laser field to electron subsystem:

10 $$DED=\underset{-\infty }{\overset{+\infty }{\int }}\left(W\left(I\right)\left(1-\frac{\rho }{{\rho }_{at}}\right)K+{W}_{1pt}\rho \right)\hslash {\omega }_{0}dt,$$

where W(I) is the field ionization probability calculated with the Keldysh’s formula^37^, ρ_at_ is the density of atoms, K is the multiphoton order. An analysis of deposited energy density achieved during laser pulse action allows detecting potential micromodification formation.

### Molecular dynamics with two-temperature model

A two-temperature model (TTM) is used to simulate heat transfer through and between electronic and atomic subsystems^35,38^. The atomic subsystem is simulated using classical MD algorithms with a classical force field (Tersoff^39^ in our case), while the electronic subsystem is modeled as a continuum or background”gas”on a regular grid that overlaps the simulation area. Energy can be transferred spatially within an electron grid. and between the electronic and atomic subsystems. Heat transfer between the subsystems is performed using a Langevin thermostat^40^. As the initial conditions, the electron density and electron temperature are set. We assumed the Gaussian distribution of electron density (maximal is 0.16 ρ_at_) and temperature (8 eV) with a FWHM of 1/10 of the simulation box`s size in x,y plane and 1/5 of the simulation box`s size in the z plane (z is an optical axis). To represent the heat transfer to the environment, we fixed the temperature (300 K) of a thin layer (5 atomic layers) of atoms in each boundary using a Langevin thermostat. The used in the simulation parameters are presented in the table below. The source code of^38^ was slightly modified to achieve the non-constant distribution of electron density and temperature. We used periodic boundary conditions and simulation 23 × 23 × 23 nm simulation cell. The used parameters for TTM model si presented in Table 1.

**Table 1:** Parameters for TTM model.

Variable name	Units	Physical meaning	Value
a_0	eV K^−1^	Polynomial fitting coefficient of electron specific heat	0.005
a_1	eV K2^−1^	Polynomial fitting coefficient of electron specific heat	0
a_2	eV K3^−1^	Polynomial fitting coefficient of electron specific heat	0
a_3	eV K4^−1^	Polynomial fitting coefficient of electron specific heat	0
a_4	eV K5^−1^	Polynomial fitting coefficient of electron specific heat	0
C_0	eV K^−1^	Polynomial fitting coefficient of electron specific heat	0
A	1 K^−1^	Exponential fitting coefficient of electron specific heat	0
Rho_e_max	Nat	Plasma electron density	0.16
D_e	A2 ps^−1^	Thermal diffusion coefficient	32,436
gamma_p	g mol^−1^ ps^−1^	friction coefficient due to electron–ion interactions	11.235
Gamma_s	g mol^−1^ ps^−1^	friction coefficient due to electronic stopping	8.443
V_0	A ps^−1^	electronic stopping critical velocity	79.76
I_0	eV ps^−1^ A^−2^	Laser intensity	0
Lsurface	–	The initial borders of vacuum	0
Rsurface	–	The initial borders of vacuum	40
L_skin	A	Skin layer depth	2
Tau	ps	Laser pulse duration	0.1
B	–	Constant of electron blast force	60
Lambda	A	electron mean free path	2
N_ion	A-3	Ion concentration	0.05
Surface_movement	–	disabled	0
T_e_min	–	Minimal electron temperature	0

## Supplementary Information

Supplementary Video 1.

## Data Availability

Data is available upon reasonable request from the corresponding author, FP.

## Code availability

Code is available upon reasonable request.

## References

[1] Pavlov I (2017). Optical waveguides written deep inside silicon by Femtosecond Laser. Opt. InfoBase Conf. Pap..

[2] Zavedeev EV, Kononenko VV, Konov VI (2016). Delocalization of femtosecond laser radiation in crystalline Si in the mid-IR range. Laser Phys..

[3] Marcinkevičius A, Mizeikis V, Juodkazis S, Matsuo S, Misawa H (2003). Effect of refractive index-mismatch on laser microfabrication in silica glass. Appl. Phys. A Mater. Sci. Process..

[4] Mareev EI (2019). Effect of pulse duration on the energy delivery under nonlinear propagation of tightly focused Cr: Forsterite laser radiation in bulk silicon. Laser Phys. Lett..

[5] Kononenko VV, Konov VV, Dianov EM (2012). Delocalization of femtosecond radiation in silicon. Opt. Lett..

[6] Verburg P (2015). Laser-Induced Subsurface Modification of Silicon Wafers.

[7] Malinauskas M (2016). Ultrafast laser processing of materials: From science to industry. Light Sci. Appl..

[8] Chanal M (2018). Crossing the threshold of ultrafast laser writing in bulk silicon. Nat. Commun..

[9] Mori M (2015). Tailoring thermoelectric properties of nanostructured crystal silicon fabricated by infrared femtosecond laser direct writing. Phys. Status Solidi Appl. Mater. Sci..

[10] Wang A, Das A, Grojo D (2020). Temporal-contrast imperfections as drivers for ultrafast laser modifications in bulk silicon. Phys. Rev. Res..

[11] Potemkin FV (2016). Overcritical plasma ignition and diagnostics from oncoming interaction of two color low energy tightly focused femtosecond laser pulses inside fused silica. Laser Phys. Lett..

[12] Richter RA (2020). Sub-surface modifications in silicon with ultra-short pulsed lasers above 2 µm. J. Opt. Soc. Am. B.

[13] Chambonneau M (2019). Competing nonlinear delocalization of light for laser inscription inside silicon with a 2-μm picosecond laser. Phys. Rev. Appl..

[14] Chambonneau M (2021). In-volume laser direct writing of silicon—Challenges and opportunities. Laser Photonics Rev..

[15] Chambonneau M (2021). Transverse ultrafast laser inscription in bulk silicon. Phys. Rev. Res..

[16] Migal E, Mareev E, Smetanina E, Duchateau G, Potemkin F (2020). Role of wavelength in photocarrier absorption and plasma formation threshold under excitation of dielectrics by high-intensity laser field tunable from visible to mid-IR. Sci. Rep..

[17] Marcinkevičiūtė A (2019). Femtosecond filamentation and supercontinuum generation in bulk silicon. Opt. Lett..

[18] Medvedev N, Rethfeld B (2010). A comprehensive model for the ultrashort visible light irradiation of semiconductors. J. Appl. Phys..

[19] Sundaram SK, Mazur E (2002). Inducing and probing non-thermal transitions in semiconductors using femtosecond laser pulses. Nat. Mater..

[20] Vailionis A (2011). Evidence of superdense aluminium synthesized by ultrafast microexplosion. Nat. Commun..

[21] Potemkin FV (2017). Enhancing nonlinear energy deposition into transparent solids with an elliptically polarized and mid-IR heating laser pulse under two-color femtosecond impact. Laser Phys. Lett..

[22] Hu Q (2011). Dynamics of Melt-Mediated Crystallization.

[23] Mareev, E. I. *et al.* A comprehensive approach for characterisation of the deposited energy density during laser-matter interaction in liquids and solids. *Meas. Sci. Technol.*10.1088/1361-6501/ab808b.

[24] Wang, A., Das, A., Hermann, J. & Grojo, D. Three-dimensional luminescence microscopy for quantitative plasma characterization in bulk semiconductors. *Appl. Phys. Lett.* **119**, (2021).

[25] Naumov AN, Sidorov-Biryukov DA, Fedotov AB, Zheltikov AM (2001). Third-harmonic generation in focused beams as a method of 3D microscopy of a laser-produced plasma. Opt. Spectrosc..

[26] Mareev, E. I., Migal, E. A. & Potemkin, F. V. Ultrafast third harmonic generation imaging of microplasma at the threshold of laser-induced plasma formation in solids. *Appl. Phys. Lett.* **114**, (2019).

[27] Mareev EI, Migal EA, Potemkin FV (2019). Ultrafast third harmonic generation imaging of microplasma at the threshold of laser-induced plasma formation in solids. Appl. Phys. Lett..

[28] Potemkin F (2017). Controlled energy deposition and void-like modification inside transparent solids by two-color tightly focused femtosecond laser pulses. Appl. Phys. Lett..

[29] Rethfeld B (2006). Free-electron generation in laser-irradiated dielectrics. Phys. Rev. B.

[30] Kaiser A, Rethfeld B (2000). Microscopic processes in dielectrics under irradiation by subpicosecond laser pulses. Phys. Rev. B Condens. Matter Mater. Phys..

[31] Christensen BH, Balling P (2009). Modeling ultrashort-pulse laser ablation of dielectric materials. Phys. Rev. B Condens. Matter Mater. Phys..

[32] Bauerhenne B, Zijlstra ES, Garcia ME (2017). Molecular dynamics simulations of a femtosecond-laser-induced solid-to-solid transition in antimony. Appl. Phys. A Mater. Sci. Process..

[33] Singh N (2010). Two-temperature model of nonequilibrium electron relaxation: A review. Int. J. Mod. Phys. B.

[34] Mareev EI, Potemkin FV (2022). Dynamics of ultrafast phase transitions in (001) Si on the shock-wave front. Int. J. Mol. Sci..

[35] Norman GE, Starikov SV, Stegailov VV, Saitov IM, Zhilyaev PA (2013). Atomistic modeling of warm dense matter in the two-temperature state. Contrib. Plasma Phys..

[36] Mareev EI, Migal EA, Potemkin FV (2018). Real-time monitoring of the energy deposition under the tight focusing of femtosecond laser radiation into a bulk transparent dielectric based on third harmonic signal. JETP Lett..

[37] Keldysh LV (1965). Ionization in the field of a strong electromagnetic wave. Sov. Phys. JETP.

[38] LAMMPS documentation. https://docs.lammps.org/fix_ttm.html. Accessed 17 January 2022.

[39] Tersoff J (1988). New empirical approach for the structure and energy of covalent systems. Phys. Rev. B.

[40] Duffy DM, Rutherford AM (2007). Including the effects of electronic stopping and electron-ion interactions in radiation damage simulations. J. Phys. Condens. Matter..

